# Development and validation of objective performance metrics for robot-assisted basic surgical skills in a simulated anastomosis task using a single-port robotic system

**DOI:** 10.1007/s11701-026-03996-0

**Published:** 2026-10-03

**Authors:** Edoardo Beatrici, Valerio Santarelli, Alessio Guidotti, Francesco Pepillo, Mario de Angelis, Attilio Barretta, Francesco Cei, Nicola Frego, Marco Paciotti, Eva Vanderstraeten, Isabelle Van Herzeele, Stefano Puliatti, Geert De Naeyer, Rui Farinha, Ruben De Groote, Sij Hemal, Peter Wiklund, Simone Crivellaro, Ben Challacombe, Niki Rashidian, Alexandre Mottrie

**Affiliations:** 1 ORSI Academy, Melle, Belgium; 2 Department of Urology, IRCCS Humanitas Research Hospital, Rozzano, Italy; 3 Department of Maternal Infant and Urologic Sciences, “Sapienza” University of Rome, Policlinico Umberto I Hospital, Rome, Italy; 4 Department of Urology, European Institute of Oncology (IEO), IRCCS, Milan, Italy; 5 Department of Thoracic and Vascular Surgery, Ghent University Hospital, Ghent, Belgium; 6 Division of Urology, University of Modena and Reggio Emilia, University Hospital of Modena, Modena, Italy; 7 Department of Urology, AZORG Hospital, Aalst, Belgium; 8 Catherine and Joseph Aresty Department of Urology, USC Institute of Urology, Keck School of Medicine, CA Los Angeles, United States; 9 Department of Urology, Icahn School of Medicine at Mount Sinai, New York, NY USA; 10 Department of Urology, University of Illinois at Chicago, Chicago, IL 60607 USA; 11 Department of Urology, Guy’s and St Thomas’ NHS Foundation Trust, London, UK; 12 Department of General, HPB Surgery and Liver Transplantation, Ghent University Hospital, Ghent, Belgium; 13 Department of Maternal Infant and Urologic Sciences, “Sapienza” University of Rome, Rome, Italy

**Keywords:** Proficiency based progression, Single port, Robotic surgery, Surgical training, Basic surgical skills

## Abstract

**Introduction:**

The chicken anastomosis task is a widely adopted model for training robotic suturing and knot-tying. Aim of this study was to develop and validate objective performance metrics for this task on the Single-Port (SP) robotic platform and to establish a proficiency benchmark for training and assessment.

**Methods:**

Seven experts in robotic surgery training and Proficiency Based Progression (PBP) developed the metrics for the SP chicken anastomosis task. The proposed metrics were refined among a panel of *n* = 14 participants with significant experience in PBP and/or robotics and/or the SP system, through a modified Delphi process. Construct validity was assessed by comparing recorded performances of *n* = 12 expert ( ≥100 SP RARPs) and *n* = 12 novice surgeons, blindly scored by two reviewers. Proficiency benchmarks were established based on performance distribution.

**Results:**

Delphi consensus achieved 100% agreement on the proposed performance metrics (*n* = 5 steps, *n* = 20 errors, *n* = 1 failure to progress and *n* = 4 critical errors [CE]). Novices were twice more likely to commit a CE (*n* = 6 vs. *n* = 3). Among participants who completed the task without CEs, novices required significantly longer completion times (mean 25.3 min, 95%CI 18.0–32.7 vs. 11.2 min, 95%CI 8.5–13.9; *p* = 0.003) and committed a significantly higher mean number of errors (12.3, 95%CI 8.6–16.1 vs. 8.1, 95%CI 6.6–9.6, *p* = 0.033). Calculated Inter-Rater Reliability (IRR: agreements/agreements+disagreements) was 0.96. Proficiency benchmarks were defined as: absence of CEs and < 10 errors.

**Conclusions:**

The proposed metrics showed robust construct validity and reliability, and support PBP training and assessment for the SP platform. The proposed benchmark, as well as its clinical impact, requires confirmation and prospective evaluation.

## Introduction

The advent of robotic assisted surgery has revolutionized clinical practice. Advantages over standard open and laparoscopic approaches include enhanced dexterity and superior visualization of the surgical field [1–4]. Robotic surgical training remains largely unstructured: learning curves start directly in the Operating Room (OR) with direct exposure to patients in most cases, without prior possibility of familiarizing with the robotic system and progressively acquiring basic surgical skills (BSS) and procedural skills [5]. This way of progressing, the so-called Halstedian model, which results in inequitable preparation of trainees, may have negative implications on safety and clinical outcomes of patients, and is considered unethical nowadays. The Proficiency Based Progression (PBP) methodology, which relies on a step-by-step proficiency-driven approach in which trainees must meet predefined performance benchmarks before advancing, has demonstrated higher performance outcomes and trainees’ satisfaction [6, 7]. In order to establish a structured PBP training pathway, objective performance metrics need to be developed on standardized training models [8–10]. The first step towards efficient procedural training relies on the acquisition of BSS, such as suturing, knotting, coagulating and dissecting. The “Venezuelan chicken model” is a low-cost, widely available, reproducible and validated training model for multiport (MP) robotic suturing and knot-tying [11–13]. Since receiving CE mark approval in 2024, the adoption of the Da Vinci^®^ (Intuitive surgical Inc., Sunnyvale, CA, USA) Single-Port (SP) robotic system across European institutions is rapidly increasing [14]. The fully articulating 3D-HD endoscope and the multi-jointed instruments allow various surgeries to be performed in narrow spaces, with high dexterity and minimized conflicts [15, 16]. Despite a similar user interface and master controllers maneuverability compared to the MP system, the different instrument handling and the narrower workspace demand a dedicated training pathway, with the need to define specific performance metrics and tailored validated benchmarks [16].

We here aimed to develop performance metrics for the Venezuelan chicken anastomosis model for the SP robotic system, in order to establish dedicated PBP training pathways for this robotic platform.

## Materials and methods

The study was reviewed and approved by the AZORG Ethics Committee, Aalst, Belgium (reference IRB no. 2025 − 178).

### Model preparation and anastomosis technique

Puliatti et al. developed and validated the performance metrics for the chicken anastomosis task on the Venezuelan chicken model with the MP robotic platform [13]. The chicken anastomosis task can be employed on any currently available robotic platform. Chicken model preparation follows a standardized protocol [13]. After having removed all abdominal organs, with the exception of the stomach (surrogate for the bladder) and 6–8 cm of cloaca (surrogate for the urethra), the model is preserved frozen until the day of use. For thawing, water at a temperature of 60 °C is run on the surface and internal cavity of the chicken for 1 min. The chicken is then set to rest at room temperature for about 1 h. The anterior abdominal wall and lower thorax are removed and the cloaca and stomach are cleansed from any residues. The cloaca is then cut to achieve an overlap with the stomach of approximately 1 cm.

### Robot set-up

For the anastomosis task, the robot is positioned with the 25 mm SP cannula at a 45° orientation relative to the operating table, the articulating camera is then positioned at 12 o’clock, and 2 SP needle drivers are installed at the 3 and 9 o’clock positions. Cadiere Forceps can be placed at the 6 o’clock position for tissue retraction. Distance between the cannula and the target anatomy should be roughly 18–20 cm. Finally, two 10 cm 3 − 0 Polysorb™ (Covidien/Medtronic, USA) sutures, pre-tied at the tail end with four knots, are employed to complete the anastomosis with the previously described Van Velthoven technique [17].

### Metrics development

The development of the performance metrics followed a methodology previously applied in the design of metrics based on the PBP methodology [13]. A steering committee comprising seven experts (A.M., E.B., E.V., N.R., P.W., R.F., S.C.), all with extensive experience in robotic surgery training and PBP methodology, developed the initial draft of the metrics. Procedural steps, as well as general and critical errors (CEs), were systematically identified and defined through internal consensus.

A modified Delphi meeting was subsequently conducted remotely on October 22, 2025. Seven panelists with extensive expertise in PBP methodology and/or SP system, and/or robotic surgery participated alongside the seven steering committee members. Panelists were asked to evaluate and vote on the predefined set of metrics, including the identification and definition of general errors for each procedural step, as well as the definition of CEs. Agreement was predefined as ≥ 80%. In cases where consensus was not reached, structured discussions were undertaken to refine or modify the definitions, followed by a subsequent round of voting. Consensus was reached after two rounds. Two of the suturing errors initially proposed by the steering committee did not reach the predefined threshold and were removed after discussion. The revised metrics set was submitted to a second voting round. The voting process was conducted using the Vevox online polling platform (Manchester, UK), which enabled real-time visualization and immediate aggregation of responses.

### Construct validity

Construct validity was assessed by comparing performance scores between experts and novices during recorded SP chicken anastomosis tasks, using the performance metrics developed and approved through the consensus process. All procedures were anonymized prior to scoring.

Videos were evaluated by two independent reviewers (A.G., F.P.) with expertise in robotic surgery and PBP methodology. Prior to formal assessment, reviewers underwent a structured training phase using separate practice videos, under the supervision of a third expert (R.F.), in accordance with previously described methodology [13]. During this phase, representative recordings of SP chicken anastomosis tasks were jointly reviewed to demonstrate the application of each metric and to clarify potential ambiguities. Reviewers then performed independent scoring on these training videos, followed by structured discussions to compare results and resolve discrepancies. This process was repeated iteratively until a predefined level of agreement between raters, calculated as the proportion of agreements over total number of ratings (agreements + disagreements), of ≥ 0.8 was achieved.

Only after reaching this threshold did reviewers proceed to the evaluation of the study videos. All study videos were assessed independently, with reviewers blinded to both participant identity and level of expertise (expert vs. novice), ensuring an unbiased evaluation process.

### Study participants

Experts were defined as surgeons with substantial experience in robotic surgery, having performed at least 100 robot-assisted radical prostatectomies (RARPs) using the SP system as the primary operator. Novices were defined as surgeons without prior experience with the SP system, who had not undergone PBP training in BSS on the MP platform, and who had performed fewer than five complete MP robot-assisted procedures as console surgeons. All participants were required to review a standardized instructional video and familiarize with the system prior to performing the task. The task consisted of constructing a vesicourethral anastomosis. Participants were instructed to place four sutures on the posterior wall (two using each of the pre-tied needles), followed by suturing of the lateral walls, beginning from either the right or left side at their discretion. Subsequently, a minimum of two sutures per needle was required on the anterior wall, after which the sutures were tied to complete the anastomosis. Exclusion criteria included any physical, cognitive, or technical limitation that could impair the participant’s ability to adequately perform robotic suturing and knot-tying.

### Statistical analysis

In the original study on the development and validation of the chicken anastomosis task on the MP robotic system, construct validity was effectively demonstrated with ten experienced and nine novice surgeons [13]. In light of the absence of prior data on SP-specific BSS and to account for potential differences and confounders introduced by the adoption of the SP platform, a sample size of 12 participants per group was deemed sufficient for our study to ensure adequate statistical power.

Descriptive statistics were employed to summarize baseline characteristics of the study participants and objective task performances. Categorical variables were reported as absolute numbers and percentages, and mean plus 95% Confidence Interval (CI) was calculated for continuous data. Outcomes were reported stratified by expertise level (i.e. experts vs. novices). An exploratory subanalysis restricted to participants who completed the procedure without critical errors (CE = No) was also performed, in case a CE (for example early breaking of the suture) resulted in early termination of the task, potentially precluding the detection of additional errors in subsequent steps and artificially reducing total task completion time. Comparisons between groups (novices vs. experts participants) were performed using the Fisher’s Exact Test or a Chi Square test for categorical data, and either the Mann-Whitney test or the ANOVA one-way analysis of variance for continuous variables. The Inter-Rater Reliability (IRR) was calculated for all participants (i.e. agreements/agreements + disagreements), referring to the scoring of the number of errors and critical errors recorded for each performance.

## Results

### The Delphi consensus

Overall, *n* = 14 participants took part in the Delphi consensus meeting. All panelists had extensive expertise in robotic surgery and/or PBP methodology and metrics development and/or the SP system. The panel comprised *n* = 12 urologists, *n* = 1 hepatobiliary surgeon and *n* = 1 veterinarian. Of these, *n* = 6 were from Italy, *n* = 4 from Belgium, *n* = 1 from Poland, *n* = 2 from the United States and *n* = 1 from Portugal.

The voting process commenced with the definition of the robotic setup. Panelists agreed that the camera should be positioned at the 12 o’clock position in “Cobra” mode, with allowance for deviation to a straight camera configuration (100% agreement). Two needle drivers were selected as the standard instruments, to be positioned at the 3 and 9 o’clock positions (100% agreement). Cadiere Forceps could be placed at the 6 o’clock position for tissue retraction.

**Fig. 1:**
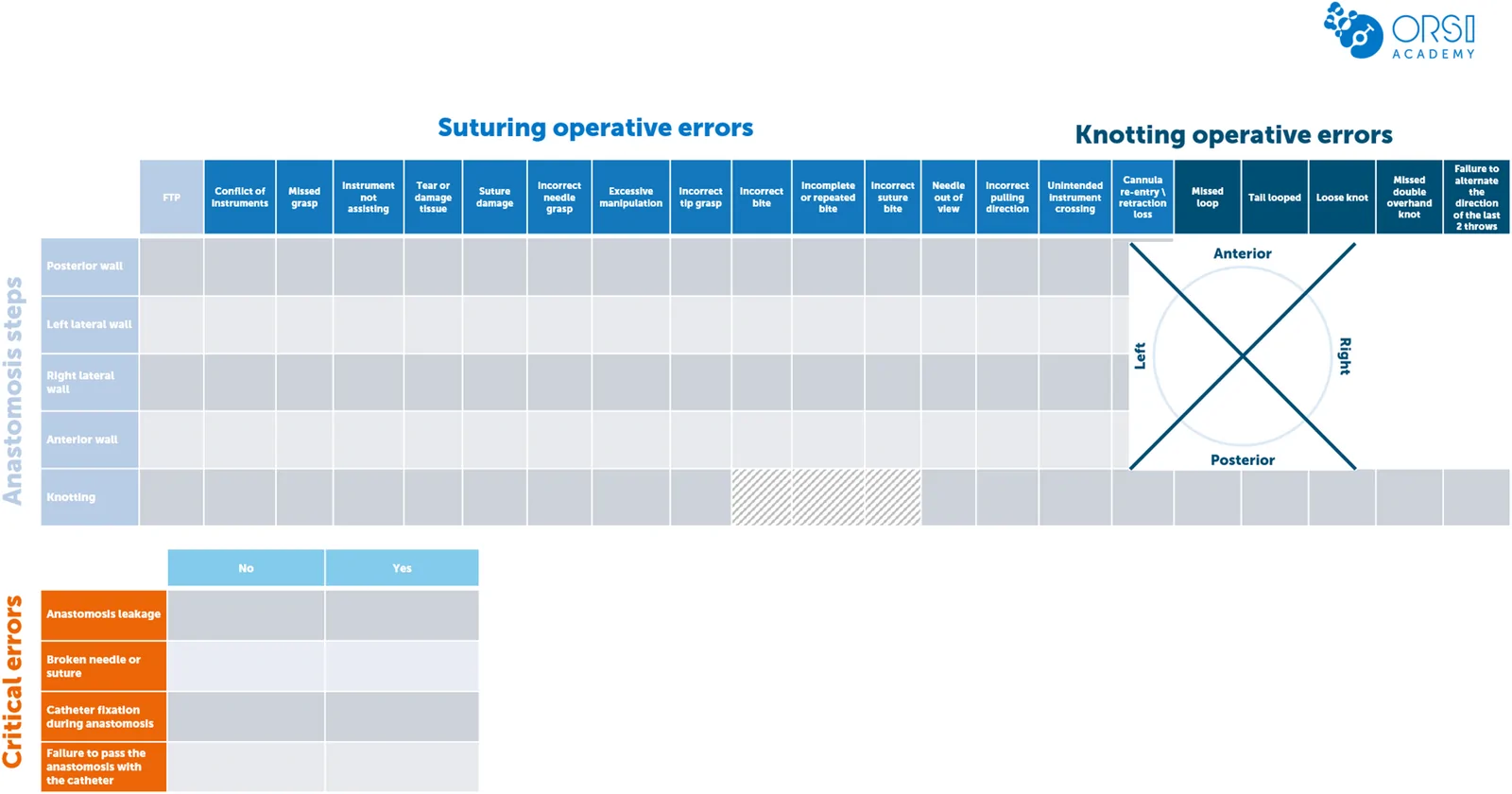
Final Scoresheet for the Single Port Chicken Anastomosis Task. FTP: Failure To Progress

The discussion then focused on defining the procedural steps. Consensus was reached on five key steps of the task: posterior wall, left lateral wall, right lateral wall, anterior wall, and knot tying (100% agreement). Panelists subsequently evaluated and voted on the list of general errors and their definitions, as well as on CEs. Two of the *n* = 17 suturing errors initially proposed did not reach the agreement threshold in the first round of voting and were removed after structured discussion, leaving *n* = 15 suturing operative errors. In addition, *n* = 5 knot-tying errors were identified, for a total of *n* = 20 errors. Consensus was also achieved on the definition of failure to progress (100% agreement). Finally, *n* = 4 CEs were identified and defined, with full agreement among panelists. Table 1 summarizes the number of steps, errors, and CEs before and after the modified Delphi consensus process, while Fig. 1 presents the resulting final scoresheet derived from this process.

**Table 1:** Number of steps, errors and critical errors before and after the modified Delphi meeting

	*N*. before Delphi	*N*. after Delphi	Agreement (%)
Steps	5	5	100
Suturing operative errors	17	15	100
Knotting operative errors	5	5	100
“Fail to progress” definition	1	1	100
Critical errors	4	4	100

### Construct validity

A total of *n* = 24 (*n* = 12 experts and *n* = 12 novices) surgeons participated in the construct validity phase and performed the chicken anastomosis task. Results stratified by surgeon experience are reported in Table 2. Mean (95% CI) time to completion of the task was significantly longer in the novice surgeons group (23.8 [18.9–28.7] min vs. 12.1 [9.3–14.9] min for novices and experts respectively, *p* < 0.001). Novices were twice more likely to incur CEs during task completion (*n* = 6 [3 anastomosis leakage, 2 broken sutures, 1 catheter fixation] vs. *n* = 3 [3 anastomosis leakage]), but the difference did not reach the level of significance (*p* = 0.400). Finally, the mean (95%CI) number of errors was 10.7 (7.8–13.5) and 8.1 (7.0–9.2) for novices and experts, respectively (*p* = 0.084). When taking into account only those participants who completed the task without committing CEs (*n* = 6 for novices and *n* = 9 for experts), the difference in mean number of errors increased, reaching the level of significance (12.3 [8.6–16.1] vs. 8.1 [6.6–9.6] respectively for novices and experts, *p* = 0.033). Similarly, among those who did not incur any CEs, novices required more than twice the time of experts to complete the exercise (25.3 [18.0–32.7] min for novices and 11.2 [8.5–13.9] min for experts, *p* = 0.003). The calculated overall IRR was 0.96 (0.92 for novices and 1 for experts).

**Table 2:** Results of the SP chicken anastomosis suturing and knot tying task stratified by surgeon experience

Variable	Total (*n* = 24)	Novices (*n* = 12)	Experts (*n* = 12)	*p*-value
Critical Errors, n (%)	9 (37.5)	6 (50.0)	3 (25.0)	0.400
Errors, mean (95% CI)	9.4 (7.9–10.9)	10.7 (7.8–13.5)	8.1 (7.0-9.2)	0.084
Time (min), mean (95% CI)	18.0 (14.3–21.6)	23.8 (18.9–28.7)	12.1 (9.3–14.9)	< 0.001
IRR, agreements/total	23/24 (0.96)	11/12 (0.92)	12/12 (1)	

Subgroup without CE	Total (*n* = 15)	Novices (*n* = 6)	Experts (*n* = 9)	*p*-value
Errors, mean (95% CI)	9.8 (7.9–11.7)	12.3 (8.6–16.1)	8.1 (6.6–9.6)	0.033
Time (min), mean (95% CI)	16.9 (12.0-21.7)	25.3 (18.0-32.7)	11.2 (8.5–13.9)	0.003

*CE* Critical Errors; *CI*: Confidence Interval; *IRR*: Inter-Rater Reliability

### Benchmark definition

**Fig. 2:**
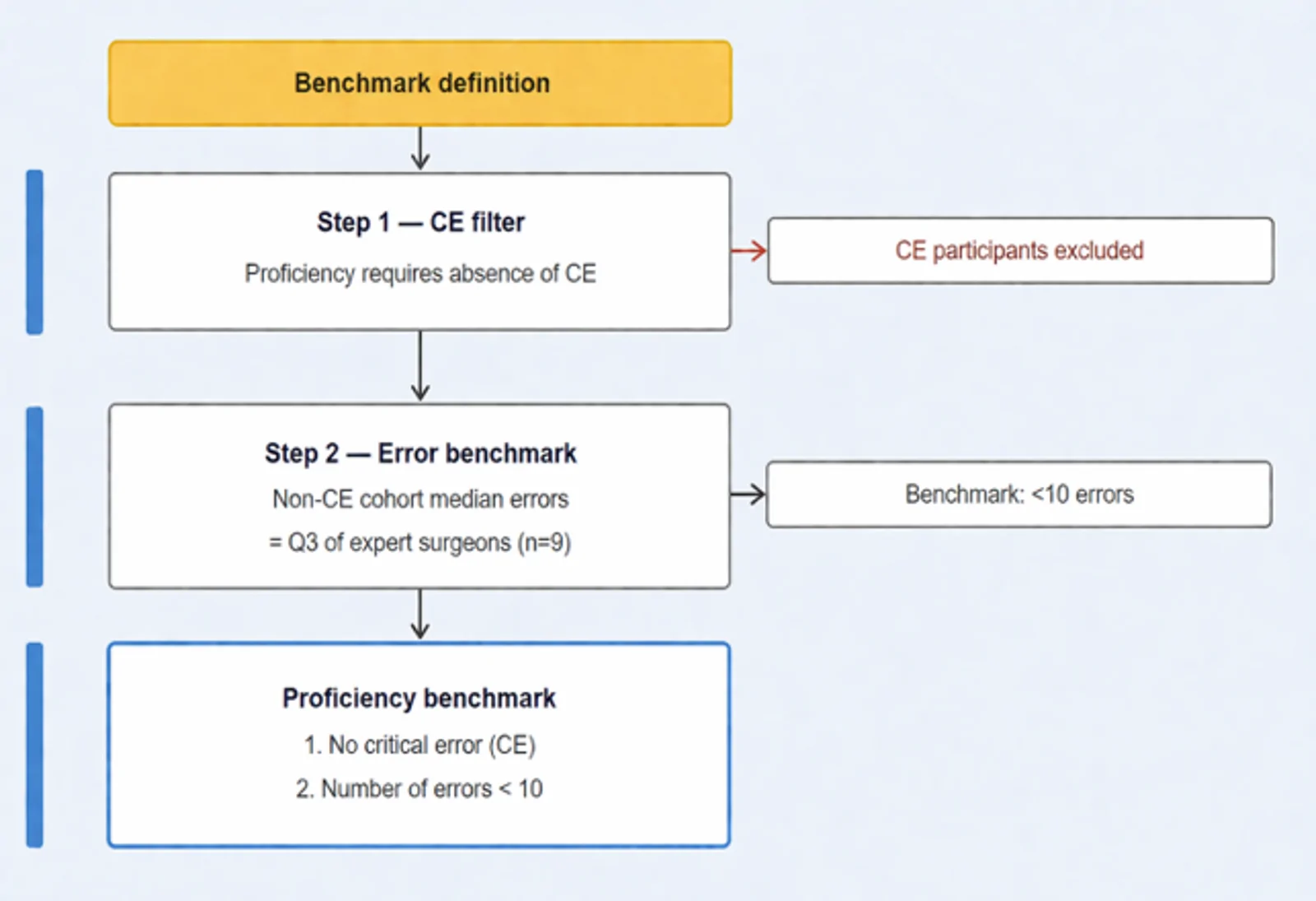
Two-step framework for proficiency benchmark definition for the SP chicken anastomosis task. CE: critical error; Q3: third quartile

Benchmark definition for proficiency achievement of the chicken anastomosis task with the SP robotic system followed two consecutive steps (Fig. 2). First, not performing a CE is required to achieve proficiency as CEs are defined as procedural deviations that, if performed during surgical procedures, would result in an irreversible adverse event or pose a direct risk to patient safety. Accordingly, only participants who did not incur CE were included to define the subsequent benchmark definition steps. Second, the median number of errors performed by the non-CE study cohort, corresponding to the third quartile for expert surgeons (*n* = 9), was identified as the benchmark for number of errors. Consequently, participants would be required to successfully complete the task without incurring any CEs and performing fewer than 10 errors to be regarded as proficient.

## Discussion

The introduction of the SP platform has been the greatest hardware innovation since the early phases of robotic surgery. Its main advantage lies in the increased maneuverability and space-efficiency provided by the multi-jointed instruments, inserted through a single surgical incision [16]. In addition to superior pain control and cosmetic results, these capabilities translate into a shift towards less invasive approaches, such as extraperitoneal routes. Avoiding critical structures within the peritoneal cavity and maintaining a standardized supine position for different urological surgical procedures has demonstrated to reduce the risk of perioperative complications [18–22].

This study developed and validated objective performance metrics for a simulation-based anastomosis task using the SP robotic platform. The most important finding is that these metrics demonstrated strong construct validity, reliably discriminating between expert and novice performance based on critical errors, completion time, and total errors. To our knowledge, this represents the first validated simulation-based assessment framework for basic surgical skills on the SP robotic system, and it provides a preliminary proficiency benchmark for future PBP training and assessment.

In the first part of our study, metrics for the SP chicken anastomosis task were adapted and validated from the MP platform by a panel of *n* = 14 experts in a modified Delphi fashion. The majority of the suture and knotting operative errors already validated for the MP system were deemed valid for the SP platform by the panel of experts. Overall, *n* = 3 new operative errors were added in the final version of the metrics. One, incorrect pulling direction, refers to the vector along which the suture can safely be pulled, which is perpendicular to the exit point of the suture. The other two are specific to the SP system: unintended instrument crossing, a consequence of the higher risk of conflict resulting from deploying the instruments and camera from a single cannula; and cannula re-entry with loss of retraction, which has no equivalent in the MP platform. While these results confirm the familiar interface and instrument handling of the two platforms, the SP-specific metrics supports the need for dedicated training pathways.

Since FDA approval in 2018, the initial approach to SP robotic surgery has been dominated by experienced surgeons. As expected in early technological diffusion phase, this pioneer’s period was characterized by heterogeneous training pathways, absence of structured curricula, and limited procedural standardization [14]. Despite these limitations, literature published in recent years consistently demonstrated several advantages of the SP system, such as better pain control and cosmetic results and shorter length of stay [19–22]. Nonetheless, the platform has been often criticized for the increased technical complexity and possibly longer learning curve (LC). While recently published LC studies mostly report manageable learning trajectories of procedures performed with the SP system, particularly for expert MP robotic surgeons, some concerns regarding the impact of the initial learning phase on patient outcomes arose [23–26]. As younger surgeons are trying to gain access to SP and in light of its relatively recent introduction in the European market, the safest possible adoption remains of utmost importance. Hence, development of precise PBP training pathways is fundamental to minimize the impact of the learning curve on OR efficiency (reducing operative time) and most importantly avoid its detrimental effect on patients outcomes. Definition and mastering of BSS, using a validated chicken anastomosis task, remains the first step towards procedural proficiency prior to practice on real patients.

The ability of the Delphi-defined metrics in this study to efficiently differentiate between experts and novices performances, has demonstrated construct validity. Particularly, novices were twice as likely to incur CEs, resulting in a failed exercise. When only considering participants who did not perform CEs, novice surgeons committed significantly more errors than expert surgeons, confirming the ability of the proposed metrics to define performance quality. Moreover, the high inter-rater agreement (0.96) suggests that the metrics can be applied consistently by trained raters.

In recent years, the European Association of Urology Robotic Urology Section (ERUS) has played a leading role in the development of structured, proficiency-based robotic procedural curricula. Such curricula are characterized by a modular step-by-step progression of increasing complexity, aiming to achieve a safe acquisition of procedural independence. Similarly, future efforts should focus on translating these validated training frameworks from the MP to the SP platform [27–29].

Our study is not without limitations. First, the relatively small sample size and various surgical backgrounds of the expert surgeons, as well as the high variability of novices’ performances, may affect the reproducibility of our findings. As a consequence, the proposed benchmark was derived from a small reference cohort and should be regarded as preliminary. Second, the metrics were specifically developed with the Da Vinci SP robotic system. Accordingly, the SP chicken anastomosis task and related metrics may not adapt to different SP platforms that are currently being introduced into the European market. Third, benchmark definition was strictly dependent on this limited surgical cohort, thus limiting generalizability of our results. Fourth, the members of the steering committee that drafted the initial metrics also participated in the Delphi voting; although consistent with the methodology adopted in previous consensus processes on this task, this may have biased the panel towards acceptance of the proposed items and reduced the independence of the consensus.

Finally, the impact of proficiency achievement for the SP chicken anastomosis task on clinical practice has not been evaluated.

## Conclusion

Objective performance metrics for the SP robotic chicken anastomosis task were developed through consensus and showed construct validity by discriminating expert from novice performance. These metrics allowed the proposition of a preliminary proficiency benchmark and support integration of the task into SP-specific PBP training pathways. Future studies should assess their impact on operative performance and patient outcomes.

## Data Availability

No datasets were generated or analysed during the current study.
